# Ontogenetic Alterations in Molecular and Structural Correlates of Dendritic Growth after Developmental Exposure to Polychlorinated Biphenyls

**DOI:** 10.1289/ehp.9773

**Published:** 2007-01-16

**Authors:** Pamela J. Lein, Dongren Yang, Adam D. Bachstetter, Hugh A. Tilson, G. Jean Harry, Ronald F. Mervis, Prasada Rao S. Kodavanti

**Affiliations:** 1 Center for Research on Occupational and Environmental Toxicology, Oregon Health & Science University, Portland, Oregon, USA; 2 Neurostructural Research Labs, Tampa, Florida, USA; 3 Center of Excellence for Aging and Brain Repair and Department of Neurosurgery, University of South Florida College of Medicine, Tampa, Florida, USA; 4 Cellular and Molecular Toxicology Branch, Neurotoxicology Division, National Health and Environmental Effects Research Laboratory, Office of Research and Development, U.S. Environmental Protection Agency, Research Triangle Park, North Carolina, USA; 5 National Institute of Environmental Health Sciences, National Institutes of Health, Department of Health and Human Services, Research Triangle Park, North Carolina, USA

**Keywords:** dendritogenesis, developmental neurotoxicology, learning and memory, molecular markers, polychlorinated biphenyls

## Abstract

**Objective:**

Perinatal exposure to polychlorinated biphenyls (PCBs) is associated with decreased IQ scores, impaired learning and memory, psychomotor difficulties, and attentional deficits in children. It is postulated that these neuropsychological deficits reflect altered patterns of neuronal connectivity. To test this hypothesis, we examined the effects of developmental PCB exposure on dendritic growth.

**Methods:**

Rat dams were gavaged from gestational day 6 through postnatal day (PND) 21 with vehicle (corn oil) or the commercial PCB mixture Aroclor 1254 (6 mg/kg/day). Dendritic growth and molecular markers were examined in pups during development.

**Results:**

Golgi analyses of CA1 hippocampal pyramidal neurons and cerebellar Purkinge cells indicated that developmental exposure to PCBs caused a pronounced age-related increase in dendritic growth. Thus, even though dendritic lengths were significantly attenuated in PCB-treated animals at PND22, the rate of growth was accelerated at later ages such that by PND60, dendritic growth was comparable to or even exceeded that observed in vehicle controls. Quantitative reverse transcriptase polymerase chain reaction analyses demonstrated that from PND4 through PND21, PCBs generally increased expression of both spinophilin and RC3/neurogranin mRNA in the hippocampus, cerebellum, and cortex with the most significant increases observed in the cortex.

**Conclusions:**

This study demonstrates that developmental PCB exposure alters the ontogenetic profile of dendritogenesis in critical brain regions, supporting the hypothesis that disruption of neuronal connectivity contributes to neuropsychological deficits seen in exposed children.

The increasing prevalence of neurodevelopmental disorders, including intellectual retardation, autism, and attention deficit hyperreactivity disorder (ADHD) cannot be explained entirely by genetic mechanisms (Faraone and Khan 2006; Muhle et al. 2004; Palomo et al. 2003). This has led to an active search for environmental exposures that modulate normal neurodevelopment. From such efforts, polychlorinated biphenyls (PCBs) have emerged as a credible risk factor for neurodevelopmental disorders (Tilson and Kodavanti 1998). A recent analysis of epidemiologic data concluded that the weight of evidence indicates a negative association between developmental exposure to environmental PCB levels and measures of neuropsychological function in infancy or childhood (Schantz et al. 2003). Combined *in utero* and lactational PCB exposure correlates with decreased IQ scores, psychomotor difficulties, impaired learning and memory, and attentional deficits. Findings from experimental animal models are consistent with those in humans including deficits in learning/memory (Hany et al. 1999; Sable et al. 2006; Schantz et al. 1989; Widholm et al. 2004) and sensorimotor (Nguon et al. 2005; Powers et al. 2006; Roegge et al. 2004) functions.

The cell and molecular mechanism(s) by which PCBs derail cognitive and psychomotor development in children remain speculative. Although experimental animal and cell culture studies have identified specific signaling pathways disrupted by developmental PCB exposure [reviewed by Kodavanti (2005)], how these molecular changes relate to functional deficits has been difficult to establish, in part because of the paucity of data describing effects of PCBs on specific neurodevelopmental events. It is postulated that PCB-induced neuropsychological deficits reflect altered patterns of neuronal connectivity (Gilbert et al. 2000; Seegal 1996). A critical determinant of neuronal connectivity is dendritic morphology. The size of the dendritic arbor and the density of dendritic spines determine the total synaptic input a neuron can receive (Engert and Bonhoeffer 1999; Purves 1988) and influence the types and distribution of these inputs (Miller and Jacobs 1984; Schuman 1997; Sejnowski 1997). Dendritic morphology and synaptic wiring are refined by experience [reviewed by Grutzendler and Gan (2006); Harms and Dunaevsky 2006; LeBe and Markram 2006), and their structural plasticity is necessary for learning and memory (Hering and Sheng 2001; Leuner and Shors 2004; Sorra and Harris 2000). Subtle perturbations of temporal or spatial aspects of dendritic growth are associated with altered behavior in experimental models, and in humans. Such structural aberrations are thought to contribute to deficits observed in a variety of neurodevelopmental disorders (Huttenlocher 1991; Jagadha and Becker 1989; Rubenstein and Merzenich 2003; Zoghbi 2003).

PCB exposure modulates several factors that regulate dendritic development. In cultured neurons, PCBs alter intracellular calcium and protein kinase C signaling [reviewed by Kodavanti (2005)], whereas *in vivo* PCB exposure transiently depletes dopamine levels (Seegal 1996), alters circulating estrogen levels and estrogen-related functions (Kaya et al. 2002; Seegal et al. 2005), and interferes with thyroid hormone signaling via both thyroid hormone receptor-dependent (Bogazzi et al. 2003; Kitamura et al. 2005; Miyazaki et al. 2004) and-independent (Bansal et al. 2005; Zoeller et al. 2000) mechanisms. That PCBs may alter dendritogenesis is further suggested by recent reports that hydroxylated PCB metabolites inhibit thyroid hormone-dependent dendritic growth in primary cultures of mouse cerebellar Purkinje cells (Kimura-Kuroda et al. 2005). However, whether this occurs *in vivo* remains in question, given the lack of persistent effects on Purkinje cells in adult rats after developmental exposure (Roegge et al. 2006). The goal of this study was to test the hypothesis that developmental PCB exposure disrupts normal ontogenetic patterns of dendritic growth *in vivo*.

## Materials and Methods

### Animals and exposure

We obtained timed pregnant Long-Evans Hooded rats (~ 230 g; Charles River Laboratories, Portage, OR) on gestational day 3 (GD3; day of insemination = GD0) and housed them under regulated temperature (21 ± 2°C), relative humidity (50 ± 10%), and a 12-hr light/dark cycle. Food (Purina Lab Chow no. 5008 lactation; no. 5001 postweaning; Purina, St. Louis, MO) and water were provided *ad libitum*. All experiments were conducted according to the Institutional Animal Care and Use Committee–approved protocols of the National Health Environmental Effects Research Laboratory of the U.S. Environmental Protection Agency.

The commercial PCB mixture, Aroclor 1254 (Lot no. 124–191; purity > 99%; AccuStandard, Inc., New Haven, CT), consists primarily of *ortho*-substituted non-coplanar congeners (Kodavanti et al. 2001) and exhibits a congener profile similar to that found in human tissues, including breast milk (Hansen 1999). Dams (*n* = 15) were dosed daily (8:00–10:00 AM) by oral gavage (2 mL/kg) with either Aroclor 1254 (6 mg/kg) or vehicle (corn oil) from GD6 through postnatal day (PND) 21. No dosing occurred on PND1. Dams delivering a litter of 10–15 pups were used in the study. On PND4, litters were culled to 10 pups with a minimum of five males. Pups were weaned on PND21. The reproductive outcome, general health, and development of rats used in these studies have been previously reported (Bushnell et al. 2002; Geller et al. 2001).

### Morphometric analyses of dendritic growth

On PNDs 22 and 60, we randomly selected one male pup per litter from six litters per treatment group. Pups were euthanized, and the cerebral hemispheres and cerebella removed for Golgi staining. The hippocampal formation was stained using the Rapid Golgi protocol (Valverde 1993), and the cerebellum was stained using a modified Golgi-Cox staining protocol (Morest 1981) in tissue sections (100 μm), prepared using a sliding microtome (American Optical, New Haven, CT). All samples were coded and camera lucida drawings obtained using a Zeiss Universal brightfield microscope equipped with drawing tube and long-working distance planapochromat objectives for subsequent morphometric analyses.

Dendritic branching was quantified from camera lucida drawings of the soma and basilar dendritic arbor of six to seven CA1 pyramidal neurons randomly selected from each of five to seven brains per treatment group. Study inclusion criteria included *a*) well-impregnated neurons with no evidence of incomplete or artificial staining; *b*) blood vessels, glia, or nondescript precipitate did not obscure neuron or branches; and *c*) the cell body was located in the middle third of the thickness of the section. Neurons were selected without regard to the number of branches with cut-ends to prevent bias toward neurons with smaller dendritic arbors. The complexity of the dendritic arbor at various distances from the soma was quantified by Sholl Analysis (Sholl 1953; Uylings and van Pelt 2002). Concentric circles of 10-μm increments were centered on the soma, and intersecting dendritic branches were counted. The total number of intersections per neuron estimated the total dendritic length of the basilar dendritic tree.

The Purkinje cell dendritic field was quantified in sagittal sections by measuring the area encompassed by the dendritic arbor in six to eight randomly chosen Purkinje cells in each of 12 cerebella per treatment group. Neurons were examined across all cerebellar lobules equally between animals, with no specific attempt to distinguish unique changes between the different lobules. Study inclusion criteria included *a*) location in the cerebellum vermis; *b*) planar dendritic arbor largely parallel to the focal plane for maximum area assessment; *c*) fully stained dendritic arbors unobscured by cells or precipitate, and *d*) an intact dendritic tree with no cut dendritic segments. The area encompassing the entire dendritic arbor was quantified using Neurolucida software (Microbrightfield, Colchester, VT). The complexity of the Purkinje cell dendritic arbor was determined by centering an eyepiece reticle indexed grid scale over the Purkinje cell dendritic arbor at 1,008× magnification. The branching density was calculated as the number of dendritic branches that intersected the grid lines per 100 μm^2^.

The number of dendritic spines was determined along 30-μm-long terminal tip segments of the basilar dendritic tree of either CA1 hippocampal pyramidal neurons or Purkinje cell dendrites. Spines were counted on four to seven random terminal tips per neuron from six to seven randomly selected CA1 or Purkinje neurons from each brain. Study inclusion criteria included *a*) well-impregnated terminal tips with no artifact or overlapping precipitate, blood vessels, or other branches; *b*) terminal tip segments were not cut; and *c*) terminal tips were planar in the section, for example, parallel to the *z*-axis. Only spines that extended laterally from the segments were counted (e.g., flanking spines). Spines oriented either directly toward or away from the observer were not counted.

Soma size was determined from camera lucida drawings of CA1 pyramidal neurons (PND22 *n* = 7 rats per group; PND60 *n* = 6) and Purkinje cells (*n* = 12 rats per group at both PNDs 22 and 60). Soma area was digitized and quantified using Neurolucida software.

Although we were interested in age-related changes in dendritic structure as a function of exposure, the experiment was not designed to address this question statistically. The requirement for rapid Golgi processing resulted in two distinct times of processing (PNDs 22 and 60) to maintain the use of one cohort. Therefore, individual statistical comparisons were conducted between exposure groups at each age. Sholl data were evaluated using the Wilcoxon rank-sign test applying a highly conservative alpha level based on the number of measurements (Dawson and Trapp, 2004). Dendritic field areas, soma size, and dendritic spines were analyzed by Student *t*-test. Statistical significance was set at *p* ≤0.05.

### Quantitative reverse transcriptase polymerase chain reaction (RT-PCR) analysis of RC3 and spinophilin transcripts

We randomly selected six litters from each treatment group for molecular studies. On PNDs 4, 7, 14, 21, and 56, one male pup from each litter was euthanized; the brain excised, frontal cortex, hippocampus, and cerebellum were rapidly dissected; and samples were stored at −80°C. Total RNA was isolated (Trizol; Invitrogen, Life Technology, Carlsbad, CA), RNA amount was determined by spectrophotometry, and quality was determined by gel electrophoresis. cDNA was synthesized using Ready-To-Go You-Prime the First-Strand Beads (Amersham Biosciences, Piscataway, NJ). Primers and probes (Table 1) specific for RC3/neurogranin and spinophilin [GeneBank accession no. U22062 and AF016252, respectively (http://www.ncbi.nih.gov/GenBank); Primer Express, Applied Biosystems, Foster City, CA] were purchased from Integrated DNA Technologies, Inc. (Coraville, IA). Amplification reactions were performed in duplicate in 25 μL final volume containing TaqMan Universal PCR Master Mix (Applied Biosystems) and 25 ng cDNA and primers and probes for both target and reference (GAPDH) genes at final concentrations of 300 and 100 nM, respectively. RT-PCR was performed using a Stratagene MX3000P (Stratagene, La Jolla, CA): 10 min at 95°C followed by 40 cycles of 30 sec at 95°C, 60 sec at 55°C, and 30 sec at 72°C. The amount of target gene in experimental samples was determined by linear regression analyses using a standard curve generated for each target gene. Expression levels of target genes were normalized against endogenous GAPDH mRNA levels. Data are expressed as the fold change relative to the control group (*n* = 4–6 per treatment group). To determine statistically significant differences, data were log transformed to improve symmetry and stabilize variance, then analyzed by two-way analysis of variance (ANOVA) with main variables of treatment and age. If treatment effects were found to be significant (*p* < 0.01), differences between PCB-treated and control values were separately evaluated using *t*-tests based on the common error term from the ANOVA; resulting *p*-values were Hochberg adjusted (Hochberg and Benjamini, 1990).

## Results

### Maternal weight and pregnancy outcome

Maternal body weights during gestation and lactation, and pregnancy outcomes as determined by mean litter size (11.6 pups) and pup survival were not altered by treatment with Aroclor 1254 (6 mg/kg/day). Weight gain during lactation was slightly reduced in PCB-exposed pups; however, at PND60, body weights were not different between treatment groups. Consistent with our previous report, preweaning behavioral differences were limited to a decrease in pup reactivity to handling following PCB exposure (Bushnell et al. 2002). These previous reports considered data from animals of both sexes from the cohort used for this current study.

### Developmental PCB exposure alters dendritic growth and spine density

Golgi analysis was used to quantify dendritic length, branching complexity and spine density as indices of the structural integrity of neuronal circuitry (Engert and Bonhoeffer 1999; Kaufmann and Moser 2000). Dendritic morphology was analyzed at PND22, the day after weaning, which corresponds to the end of the exposure period, and at PND60, by which time the dendritic arbor has reached its mature size. Since learning and memory deficits occur in multiple species after exposure to PCBs (Schantz et al. 2003), we examined dendritic arborization of CA1 pyramidal neurons in the hippocampus as one primary brain region implicated in learning and memory (Squire 1992). The dendritic morphology of Purkinje cells was examined based upon the well-established vulnerability of the cerebellum to developmental hypothyroidism (Dong et al. 2005; Li et al. 2004; Thompson and Potter 2000; Zoeller and Rovet 2004).

### Pyramidal neurons of the CA1 hippocampal formation

**Figure 1:**
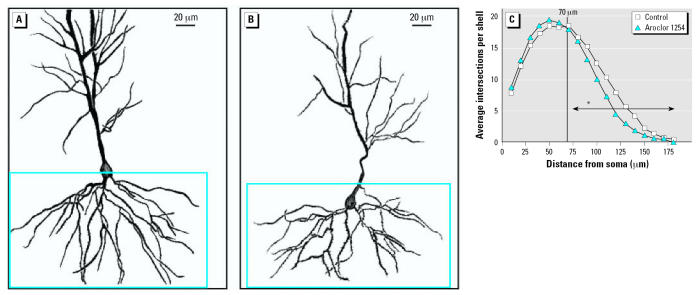
Dendritic complexity of CA1 pyramidal neurons is decreased in PND22 rat pups exposed to PCBs during gestation and lactation. Camera lucida drawings of CA1 pyramidal neurons from PND22 rat pups born to dams exposed daily to corn oil vehicle (*A*) or Aroclor 1254 at 6 mg/kg (*B*) by oral gavage from GD6 through PND21. Dendritic complexity of the basilar tree of these neurons (outlined in blue in *A* and *B*) was quantified using Sholl analysis (*C*). Statistical analysis of Sholl data indicates that the distal two-thirds of the dendritic arbor in CA1 pyramidal neurons of PND22 rats exposed to Aroclor 1254 is significantly less than that of age-matched controls (Wilcoxon test, *W* = 78, *p* < 0.005). Vertical line in *C* indicates separation from proximal to distal portion of dentritic arbor. Asterisk on horizontal arrow indicate the effect of Aroclor 1254 at *p* < 0.005.

**Figure 2:**
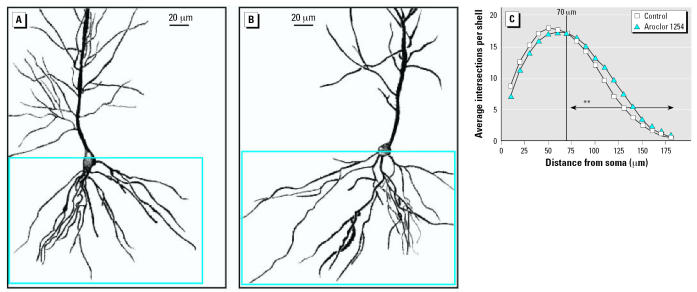
Dendritic complexity of CA1 pyramidal neurons is increased in PND60 rat pups exposed to PCBs during gestation and lactation. Camera lucida drawings of CA1 pyramidal neurons from PND60 rat pups born to dams exposed daily to corn oil vehicle (*A*) or Aroclor 1254 at 6 mg/kg (*B*) by oral gavage from GD6 through PND21. Dendritic complexity of the basilar tree of these neurons (outlined in blue in panels *A* and *B*) was quantified using Sholl analysis (*C*). Statistical analysis of Sholl data indicates that the distal two-thirds of the dendritic arbor in CA1 pyramidal neurons of PND60 rats exposed to Aroclor 1254 is significantly greater than that of age-matched controls (Wilcoxon test, *W* = 78, *p* < 0.005). Vertical line in *C* indicates separation from proximal to distal portion of dentritic arbor. Asterisks on horizontal arrow indicate the effect of Aroclor 1254 at *p* < 0.005.

Representative camera lucida drawings of hippocampal CA1 pyramidal neurons from control (Figure 1A) and PCB-exposed (Figure 1B) rats demonstrate that developmental PCB exposure decreases dendritic length and branching complexity at PND22. Sholl analysis (Figure 1C) demonstrates no significant differences between PCB-exposed and control groups in the proximal one-third of the basilar dendritic arbor. However, PCB treatment caused a significant reduction in the distal two-thirds of the basilar dendritic arbor of CA1 pyramidal neurons (*p* < 0.005). Comparison of CA1 pyramidal neurons from PCB-exposed animals (Figure 2B) versus controls at PND60 (Figure 2A) revealed a prolonged effect of developmental PCB exposure on dendritic morphology. Similar to the pattern seen in weanling rats, Sholl analysis (Figure 2C) indicates no significant difference in the proximal one-third of the basilar dendritic arbor, but differences in the distal two-thirds were characterized as a significant increase (*p* < 0.005) in the distal portion of the basilar arbor of CA1 pyramidal neurons in PCB-exposed rats relative to age-matched controls.

**Figure 3:**
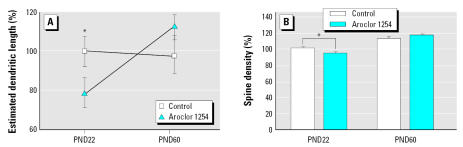
Developmental exposure to PCBs alters dendritic morphogenesis in CA1 pyramidal neurons. (*A*) Comparison of the estimated cumulative length of the distal two-thirds of the basilar trees as a function of age expressed as a percent of control at PND22. Relative to age-matched controls, the CA1 pyramidal neurons in rats exposed developmentally to Aroclor 1254 (6 mg/kg in the maternal diet) exhibit 20% less dendritic arbor at PN22 (*t* = 2.136, *p* < 0.05). While elevated by 15%, no statistical significant difference was seen in the dendritic arbor at PND60. (*B*) Quantification of dendritic spine density on the terminal tip segments of CA1 pyramidal neurons indicates that developmental exposure to Aroclor 1254 causes decreased spine density at PND22 but has no effect on spine density at PND60. Data expressed as the mean percent of age-matched control ± SE (seven rats per group and six neurons per hippocampus). *Significantly different from age-matched control (*t* = 2.835, *p* < 0.05).

Although the experimental protocol did not allow for a direct statistical comparison between ages, data from the current study were consistent with previous reports that dendritic growth of CA1 pyramidal neurons reaches adult levels by PND22 (Jacobson et al. 1988). The estimated dendritic length of the distal CA1 basilar dendritic arbor in control animals did not appear to change from PND22 to PND60, whereas an increase was suggested in the PCB-treated animals (Figure 3A). There was no evidence of a generalized hypotrophic or hypertrophic cellular response, as soma size of CA1 pyramidal neurons was not affected by PCB exposure at either age (PND22: controls, 190 μm^2^ ± 7; PCB-treated, 190 μm^2^ ± 6; PND60: controls, 178 μm^2^ ± 6; PCB- treated, 174 μm^2^ ± 8).

Analysis of spine density on the terminal tips of dendrites in CA1 pyramidal neurons (Figure 3B) shows significantly fewer spines (7% decrease, *t* = 2.835, *p* < 0.05) per unit length in the PCB-exposed rats at PND22 compared with age-matched controls. Given the general increase in dendritic spine density with age (Figure 3B), the loss of any significant differences between the two groups at PND60 suggests enhanced dendritic growth in the PCB-exposed animals from PND22 to PND60.

### Cerebellar Purkinje cells

**Figure 4:**
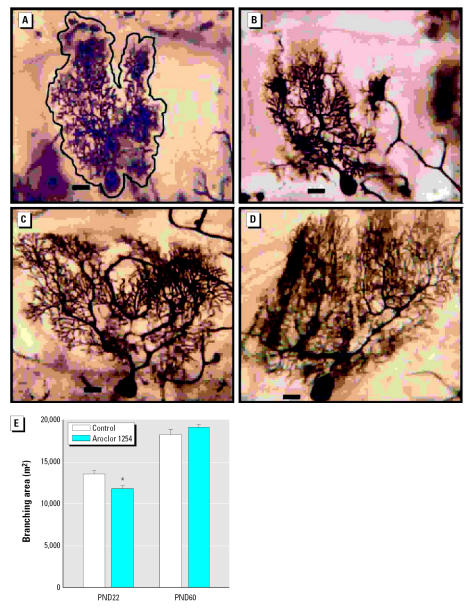
Developmental exposure to PCBs decreases the dendritic branching area of Purkinje cells at PND22, but not at PND60. Photomicrographs of representative Golgi-stained Purkinje cells from the cerebellum of PND22 (*A,B*) and PND60 (*C,D*) rat pups exposed developmentally to either vehicle (*A,C*) or Aroclor 1254 at 6 mg/kg via maternal gavage (*B,D*). (*E*) Quantitative assessment of the branching area of Purkinje cells in vehicle control and Aroclor 1254–treated rat pups as a function of age. Data are expressed as mean ± SE (*n* = 5 for control and *n* = 6 for PCB-treated rats at PND22; *n* = 5 for control and *n* = 7 for PCB-treated rats at PND60; eight neurons analyzed per cerebellum). Scale bars = 20 μm. *Significantly different from age-matched control (*t* = 3.013, *p* < 0.005).

As illustrated in photomicrographs of Golgi-stained Purkinje cells from PN22 rats (Figure 4), PCB exposure caused a 13% decrease in the area encompassing the Purkinje cell dendritic arbor relative to that in controls (Figure 4E). The size of the Purkinje cell soma was not affected by PCB exposure (PND22: controls, 406 μm^2^ ± 11; PCB-treated, 386 μm^2^ ± 9; PND60: controls, 334 μm^2^ ± 12; PCB-treated, 334 μm^2^ ± 7), suggesting that the decreased dendritic field was not due to general cellular atrophy. Consistent with previous reports (Takacs and Hamori 1994), a general maturation with an increase in the dendritic arbor and spine density was seen in both groups (Figure 4). At PND60, the difference between the two groups was no longer evident and there were no significant differences in Purkinje cell dendritic area between treatment groups (Figure 4E). This suggests a stimulation of dendritic growth in the PCB-exposed animals with the cessation of active exposure. PCB exposure did not alter age-related increases in dendritic branch density of Purkinje cells between PND22 and PND60 (PND22: controls, 49 μm^2^ ± 1.0; PCB-treated 45.5 μm^2^ ± 1.2; PND60: controls, 56.0 μm^2^ ± 1.7; PCB-treated, 57 μm^2^ ± 1.2). No significant differences were observed in spine density between controls and PCB-exposed rats at either age. However, consistent with previous data (Takacs and Hamori 1994), control groups showed a significant increase in dendritic spine density between PND22 and PND60 (Figure 4).

### Developmental exposure to PCBs alters the ontogenetic pattern of RC3/neurogranin and spinophilin mRNA levels

In conjunction with Golgi analysis, we employed quantitative RT-PCR to examine time- and region-specific effects of PCBs on spinophilin and RC3/neurogranin expression in the hippocampus, cerebellum, and cortex. Spinophilin, an actin-binding protein localized primarily to dendritic spines (Satoh et al. 1998; Allen et al. 1997) is implicated in spine maturation (Terry-Lorenzo et al. 2005) and used as a marker of spine density in many brain regions, particularly the hippocampus (Law et al. 2004; Li et al. 2004; Ouimet et al. 2004). RC3/neurogranin is a brain-specific protein kinase C substrate involved in the regulation of calcium signaling and neuronal plasticity (Pak et al. 2000) and is expressed at high levels in the somatodendritic domain of neurons (Neuner-Jehle et al. 1996; Represa et al. 1990; Watson et al. 1994). RC3/neurogranin has been proposed as a molecular indicator of dendritic arborization (Li et al. 2000), and its expression at the mRNA level has been demonstrated previously to be sensitive to developmental PCB exposure (Zoeller et al. 2000).

**Figure 5:**
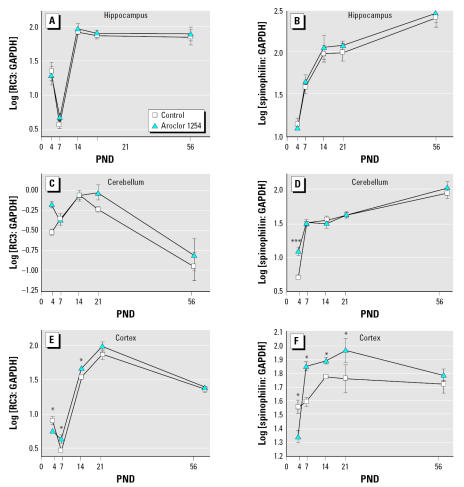
Developmental exposure to PCBs alters RC3/neurogranin (*A,C,E*) and spinophilin (*B,D,F*) mRNA levels in the developing rat brain. Total RNA was isolated from the hippocampus (*A,B*), cerebellum (*C,D*), and frontal cortex (*E,F*) of rat pups at varying ages after birth to dams exposed to vehicle or Aroclor 1254 (6 mg/kg) daily via gavage from GD6 through PND21. RC3/neurogranin, spinophilin, and GAPDH mRNA were determined using real-time RT-PCR. RC3/neurogranin and spinophilin mRNA values were normalized against endogenous GAPDH mRNA levels, log-transformed and expressed as mean ± SE. Transcript levels were analyzed by two-way ANOVA followed by post hoc analysis of differences at each time point by *t*-test. *p < 0.05, ***p < 0.001.

Regional differences were observed in the developmental profile of both spinophilin and RC3/neurogranin mRNA levels during the period of extensive and rapid dendritic growth (the first 3 weeks after birth) and in the adult brain. In control animals, levels of spinophilin mRNA steadily increased with age from PND4 to PND56 in the hippocampus and cerebellum and from PND4 to PND21 in the cortex (Figure 5). Comparisons between these distinct brain regions showed that during early postnatal development, levels of spinophilin mRNA were not significantly different across all three brain regions, but with increasing age, levels of spinophilin mRNA were increasingly higher in the hippocampus relative to the cerebellum and cortex. By PND56, higher levels of spinophilin mRNA were seen in the hippocampus compared with the other two brain regions. These data are consistent with observations that in the rat, extensive synaptogenesis occurs in these brain regions after weaning (Rice and Barone 2000) and with our Golgi analyses, showing an age-related increase in hippocampal dendritic spine density. The developmental pattern for RC3/neurogranin mRNA was more complex. In the hippocampus, RC3/neurogranin mRNA levels increased significantly during the period of rapid dendritic growth to reach maximal levels at PND14 and remained at this maximal level at PND56 (Figure 5). Similarly, in the cerebellum and cortex, RC3/neurogranin mRNA levels increased significantly during early postnatal development. In the cerebellum, the maximum level was observed at PND14; in the cortex this was at PND21. However, unlike the hippocampus, in the cortex and cerebellum, these maximal RC3/neurogranin mRNA levels were not maintained at PND56 but rather decreased to levels observed at very early postnatal ages (Figure 5). Consistent with previous reports (Singec et al. 2003), the RC3/neurogranin mRNA levels in the cerebellum were much lower relative to those detected in the hippocampus and frontal cortex at all ages.

Developmental exposure to Aroclor 1254 generally increased both spinophilin and RC3/neurogranin mRNA levels (Figure 5). With respect to RC3/neurogranin, two-way ANOVA using PCB treatment and age as main effects identified no significant differences in the hippocampus between control and PCB-treated animals. Single treatment effects were observed in the cerebellum [F(1,36) = 4.48, *p* < 0.04]. A significant interaction between age and treatment was detected in the cortex [F(4,38) = 4.98, *p* < 0.003] with significant treatment differences at 4, 7, and 14 days (adjusted two-sided *p* < 0.04). Interestingly, PCB treatment decreased RC3/neurogranin levels at PND4 but increased levels at PNDs 7 and 14. With respect to spinophilin transcript levels, in the hippocampus there was a significant effect due to age [F(4,38) = 65; *p* < 0.001], but there were no significant effects because of treatment either as an interaction with age or as a main effect. In the cerebellum, an interaction between age and treatment was identified [F(4,39) = 4.10, *p* < 0.007], with the only significant difference indicated at PND4 (adjusted two-sided *p* < 0.0004). A significant interaction between age and treatment was also indicated for spinophilin levels in the cortex [F(4,37) = 4.64, *p* < 0.004). PCB treatment significantly decreased spinophilin transcript levels at PND4 (adjusted two-sided *p* < 0.05), and significantly increased spinophilin mRNA levels at PNDs 7, 14, and 21 (adjusted two-sided *p* < 0.05).

## Discussion

Our findings support the hypothesis that PCBs perturb neuronal connectivity in the developing brain by interfering with dendritogenesis. Developmental PCB exposure caused an apparent delay in dendritic growth in hippocampal CA1 pyramidal neurons at weaning as evidenced by a significant decrease in branching complexity of the distal basilar dendritic trees at PND22. However, this initial impairment of dendritic growth was followed by subsequent enhanced dendritic growth, allowing for a mature dendritic aborization that was increased relative to controls. Similar patterns of initial dendritic growth impairment followed by enhanced dendritic growth have been observed in hippocampal CA1 pyramidal neurons after developmental exposure to ethanol (McMullen et al. 1984) or application of tetanus toxin (Groc et al. 2003). The response seen in cerebellar Purkinje cells was similar although less robust. Unlike the observations made of CA1 pyramidal neurons, the dendritic area in the adult was similar between the two groups, and no PCB effects were seen on dendritic branching complexity or spine density. A recent study similarly reported that PCBs did not alter the branching density of Purkinje cells (Roegge et al. 2006). However, in contrast to our findings, previous studies reported that Aroclor 1254 (identical lot and dose) had no effect on dendritic area (Roegge et al. 2006) or dendritic length (Yang et al. 2006) in Purkinje cells using a different dosing regimen. Although we exposed pregnant dams from GD6 until PND21, both these studies initiated exposure of dams preconception, with continued exposure through PND21. Beginning PCB exposure before gestation may have changed the profile of metabolic enzyme induction, thereby altering the metabolism and excretion of PCBs. That seemingly slight difference in the timing of exposure to PCBs can affect the neurologic consequences of exposure is supported by observations of impaired radial arm maze performance in rats if exposure begins on GD6 (Roegge et al. 2000) but not if exposure begins before conception (Roegge et al. 2006).

Developmental PCB exposure also caused region-specific changes in the ontogenetic profile of transcripts encoding the dendrite-specific proteins spinophilin and RC3/neurogranin. Previous *in situ* hybridization studies (Zoeller et al. 2000) demonstrated increased RC3/neurogranin mRNA expression at early postnatal ages after developmental exposure to Aroclor 1254 at concentrations comparable to those used in this study. Based on our molecular observations, we would predict developmental PCB exposure to increase dendritic growth and/or spine formation. Although our RT-PCR analyses were not predictive of the structural outcome in the hippocampus or cerebellum at weaning, they were consistent with the normal dendritic growth that occurs with maturation and support the suggestion of enhanced dendritic growth between PNDs 22 and 60. These data also suggest that the cortex may be more sensitive to effects of PCBs on dendritic growth than either the hippocampus or cerebellum. Given the critical involvement of cortical functioning in learning and memory, Golgi analyses of cortical cell morphology is warranted; however, further refinement of a possible localized cortical target would significantly improve the ability to detect biologically meaningful changes.

The molecular and morphologic evaluations of dendritic arborization were not consistent across all ages. Previous reports, in which changes in spinophilin and RC3/neurogranin expression were found to correlate with changes in dendritic spine density or dendritic arborization (Law et al. 2004; Li et al. 2000; 2004), were based on data obtained from adult subjects. Considered with our findings, these data suggest that spinophilin and RC3/neurogranin mRNA levels may provide a good estimate of changes in dendritic growth in adult animals and may reflect changes in the rate of dendritic growth in the rapidly developing postnatal brain but may not be reliable indicators of dendritic structure during dynamic stages of dendritic arborization in the rapidly developing postnatal brain. One possible explanation of the disconnect between PCB effects on molecular markers of dendritic morphogenesis versus structural correlates of dendritic morphology at weaning, is that while PCBs increase transcription of spinophilin and RC3/neurogranin genes, translation of these markers was not increased. Based upon observations in spinophilin-deficient mice indicating that the protein is not necessary for spine formation or removal (Feng et al. 2000), one may not expect a direct correlation between mRNA and protein levels and dendritic morphogenesis. Interestingly, spinophilin and RC3/neurogranin have been implicated in the regulation of dendritic plasticity (Terry-Lorenzo et al. 2005; Pak et al. 2000), raising the possibility that although PCB effects on spinophilin and RC3/neurogranin mRNA levels do not consistently correlate with PCB effects on dendritic structure, they may be suggestive of PCB interference with dendritic or spine plasticity.

Dendritic growth and spine formation are regulated by multiple environmental cues, including hormonal status (Cooke and Woolley 2005; Kapfhammer 2004) and neuronal activity (Lohmann et al. 2005). Experimental evidence indicates that developmental PCB exposure influences each of these factors, but of these, the effects on thyroid signaling are the most extensively characterized. PCBs are known to perturb thyroid hormone function in experimental animals via reduction of circulating thyroid hormone levels or modulation of thyroid hormone signaling (Gauger et al. 2004; Zoeller et al. 2000). Given the known adverse nature of thyroid hormone dys-regulation on the developing nervous system (Thompson and Potter 2000; Zoeller and Rovet 2004), together with evidence that PCBs influence circulating levels of thyroid hormone in infants (Hagmar 2003; Koopman-Esseboom et al. 1994; Persky et al. 2001; Sala et al. 2001), this has been considered a primary mechanism for adverse neurodevelopmental effects seen in children exposed to PCBs (Hagmar 2003; Koopman-Esseboom et al. 1994; Sala et al. 2001). Neonatal hypothyroidism has been linked to dysmorphic dendritic development in the cortex (Nicholson and Altman 1972; Ruiz-Marcos et al. 1994), hippocampus (Rami et al. 1986), and cerebellum (Kimura-Kuroda et al. 2005; Nunez et al. 1992). Since a PCB exposure paradigm comparable to that used in the current study has been shown to decrease circulating thyroid hormone levels (Crofton et al. 2000) and to exert thyroidlike actions in the developing brain (Zoeller et al. 2000), as confirmed by our observations of PCB-induced upregulation of RC3/neurogranin mRNA, disruption of thyroid hormone signaling is a plausible mechanism to explain the PCB effects on both molecular and structural correlates of dendritic growth reported in the current study.

Dendritic structure is also dynamically shaped by neuronal activity, and decreased neuronal activity can result in dendritic retraction (Kater and Mills 1991). Developmental PCB exposure has been shown to interfere with neuronal activity, specifically decreasing cholinergic input (Corey et al. 1996; Provost et al. 1999), dopaminergic signaling (Seegal 1996), and glutamatergic signaling (Gafni et al. 2004). In addition, PCBs inhibit growth of afferent mossy fibers in the hippocampus (Pruitt et al. 1999). Such effects could also contribute to the impaired dendritic maturation observed in PND22 animals exposed to PCBs. The effects of neuronal activity on dendritic growth are primarily mediated by calcium-dependent signaling pathways (Lohmann et al. 2005), and studies of cultured neurons demonstrate that PCBs perturb intracellular calcium signaling pathways downstream of neuronal depolarization (Kodavanti et al. 1993; Shafer et al. 1996; Wong et al. 1997). Whether and how PCB effects on calcium signaling contribute to PCB effects on dendritic growth are difficult to predict in light of emerging evidence that calcium can either increase or decrease dendritic growth, depending on the nature of the signal (global versus local calcium changes) and the developmental stage of the neuron (Lohmann et al. 2005). Additional studies will be necessary to determine the relationships between PCB-induced perturbations of afferent input, intracellular calcium and dendritic growth. However, it seems likely that the outcome of developmental PCB exposure on dendritic morphogenesis represents the net balance between PCB effects that could enhance dendritic growth (increased spinophilin and/or RC3/neurogranin expression, enhanced thyroid hormone or estrogen signaling, moderate increases in intracellular calcium signaling) and those that potentially restrict dendritic growth (antiestrogenic effects, hypothyroidism, decreased neuronal activity consequent to reduced afferent input, or extreme increases in intracellular calcium), and this balance is likely to change as a function of the timing and duration of exposure.

In summary, our findings support the hypothesis that developmental PCB exposure disrupts neuronal connectivity as evidenced by significant alterations in dendritic structure and expression of transcripts encoding dendritic proteins in the hippocampus and cerebellum of both developing and mature brains. The hippocampus has long been implicated in learning and memory (Squire 1992), and emerging evidence indicates that cerebellar function critically influences cognitive functions as well (Hernandez-Muela et al. 2005). Given the influence of dendritic morphology on neuronal function (Engert and Bonhoeffer 1999; Purves 1988; Schuman 1997; Sejnowski 1997) and the role of dendritic plasticity in learning and memory (Hering and Sheng 2001; Leuner and Shors 2004; Sorra and Harris 2000), the dysmorphic pattern of dendritic development and maturation observed in these neuronal cell populations following developmental PCB exposure could be an important factor in the cognitive impairment observed following developmental PCB exposure.

## Figures and Tables

**Table 1:** Sequence of primers and probes used in real-time RT-PCR analyses.

RC3	
Forward primer	5′GCCAGACGACGATATTCTAGACATC–3′
Reverse primer	5′–TTTATCTTCTTCCTCGCCATGTG–3′
Probe	VIC-CCCGGAGCCAACGCCGCT-TAMRA
Spinophilin	
Forward primer	5′–AAGGCGGCCCACCATAA–3′
Reverse primer	5′–GCCCATCTGCAGGAACATACTT–3′
Probe	FAM-TATGGCTCCAACGTCCA-TAMRA
GAPDH	
Forward primer	5′–GGCACAGTCAAGGCTGAGAAT–3′
Reverse primer	5′–TCTCGCTCCTGGAAGATGGT–3′
Probe	VIC-AGCTGGTCATCAACGGGAAACCCA-TAMRA
